# Network analysis of 16S rRNA sequences suggests microbial keystone taxa contribute to marine N_2_O cycling

**DOI:** 10.1038/s42003-023-04597-5

**Published:** 2023-02-23

**Authors:** Brett D. Jameson, Sheryl A. Murdock, Qixing Ji, Catherine J. Stevens, Damian S. Grundle, S. Kim Juniper

**Affiliations:** 1 School of Earth & Ocean Sciences, University of Victoria, P.O. Box 1700 Station CSC, Victoria, BC V8W 2Y2 Canada; 2 Department of Biology, University of Victoria, P.O. Box 1700 CSC, Victoria, BC V8W 2Y2 Canada; 3 Bermuda Institute of Ocean Sciences, 17 Biological Station, St. George’s, GE01 Bermuda; 4 Thrust of Earth, Ocean & Atmospheric Sciences, Hong Kong University of Science and Technology (Guangzhou), Nansha, Guangzhou, Guangdong 511400 China; 5 School of Ocean Futures & School of Earth & Space Exploration, Arizona State University, Tempe, AZ 85287-7904 USA; 6 Ocean Networks Canada, 2474 Arbutus Road, Victoria, BC V8N 1V8 Canada

**Keywords:** Water microbiology, Element cycles, Microbial ecology

## Abstract

The mechanisms by which large-scale microbial community function emerges from complex ecological interactions between individual taxa and functional groups remain obscure. We leveraged network analyses of 16S rRNA amplicon sequences obtained over a seven-month timeseries in seasonally anoxic Saanich Inlet (Vancouver Island, Canada) to investigate relationships between microbial community structure and water column N_2_O cycling. Taxa separately broadly into three discrete subnetworks with contrasting environmental distributions. Oxycline subnetworks were structured around keystone aerobic heterotrophs that correlated with nitrification rates and N_2_O supersaturations, linking N_2_O production and accumulation to taxa involved in organic matter remineralization. Keystone taxa implicated in anaerobic carbon, nitrogen, and sulfur cycling in anoxic environments clustered together in a low-oxygen subnetwork that correlated positively with nitrification N_2_O yields and N_2_O production from denitrification. Close coupling between N_2_O producers and consumers in the anoxic basin is indicated by strong correlations between the low-oxygen subnetwork, PICRUSt2-predicted nitrous oxide reductase (*nosZ*) gene abundances, and N_2_O undersaturation. This study implicates keystone taxa affiliated with common ODZ groups as a potential control on water column N_2_O cycling and provides a theoretical basis for further investigations into marine microbial interaction networks.

## Abstract (web summary)

Network analysis of 16S rRNA-sequencing data collected over six months in the Sannich Inlet on Vancouver Island, Canada, suggests keystone microbial taxa that might contribute to water column N2O production and accumulation.

## Introduction

Linking the dynamics of complex marine microbial communities to ecosystem-scale biogeochemical processes is one of the foremost challenges in the field of microbial ecology^1,2^. The application of high-throughput gene sequencing and meta-omics to surveys of marine microbial communities has resulted in a growing awareness of the ubiquity, throughout the microbial phylogenetic tree and across a broad range of environments, of enzyme-encoding functional genes responsible for mediating core biogeochemical cycles^3,4^. Functional genes of this sort co-vary strongly with environmental variables, leading to an emerging view of microbial communities as meta-organisms rather than assemblages of interacting taxa^5,6^. From a biogeochemical point of view, proximal, abiotic controls on rate processes can now be seen as shaping genomes, transcriptomes and proteomes across environmental gradients, rather than simply modulating inputs and outputs from microbial black boxes^7–9^. This is especially true for marine oxygen deficient zones (ODZs) and sulfidic basins, where sharp redox gradients constrain the taxonomic identity and metabolic potential of microbial constituents, as well as the character of key biogeochemical transformations^7,10,11^. Robust patterns of metabolic niche differentiation have since led to the development of conceptual models that describe coupled metabolic interactions between key players across vertical redox gradients^7,12^. However, from an ecological point of view, the meta-organism paradigm provides limited insight into the mechanisms that drive community assembly at the taxonomic level, and how ecosystem function arises from a complex network of biogeochemical and ecological interactions between individual taxa.

Ecosystem function is an emergent phenomenon that results from the cumulative set of metabolic and ecological interactions that characterize dynamic and co-evolving microbial communities in the environment^13^. These interactions occur at the microscopic level and involve numerous individual taxa with diverse functional capacities. Individual cells, strains, or functional groups within a community can influence each other through a multitude of mechanisms, including mutualistic cross-feeding^14^, resource competition^15^, the production of public goods^16^ or allelopathic compounds^17^, and many others^18^. Unfortunately, ecological interactions of this sort are difficult to characterize given the cryptic nature of natural microbial communities. Complicating matters further is the fact that the vast majority of microbial organisms remain uncultured, and thus taxonomically and functionally ambiguous. Fortunately, improvements to high-throughput sequencing technologies and data processing tools combined with novel statistical approaches have allowed researchers to study patterns of microbial community assembly in unprecedented detail^19–21^.

Network analyses applied to the study of complex microbial communities has resulted in a corpus of literature documenting spatiotemporal co-occurrence patterns amongst microbial community members across a wide range of terrestrial and aquatic ecosystems^22–24^. These tools go beyond traditional assessments of biodiversity and community structure by utilizing pairwise correlations between individual taxonomic units to identify core community members and assess co-occurrences between individual taxa. Although the mechanisms driving specific co-occurrences cannot be discerned from network analyses alone, parallel information on rate processes and environmental variables can help generate testable hypotheses regarding putative ecological interactions responsible for driving ecosystem function^11^. This is a potentially fruitful line of investigation, given recent progress in linking microbial community structure to large-scale ecosystem processes such as carbon export, nitrogen fixation, and denitrification^25–27^. A holistic approach to the study of marine biogeochemical processes that encapsulates the internal complexity of entire communities may also help to elucidate ecological controls that have been previously overlooked by a reductionist focus on protein-encoding functional genes.

Empirical and computational studies suggest that microbial communities contain keystone members, defined as highly connected taxa that exert considerable influence over community structure and function irrespective of their abundances across space and time^28^. Keystone taxa can exist independently or may also be part of keystone guilds comprising several taxa of similar niche-preferences and functional properties^26^. More importantly, keystone taxa may exert influence over ecosystem processes directly, such as through the production or utilization of shared metabolites^29^, or indirectly by modulating the broader community structure^30^. This evidence suggests that ecological interactions between functionally diverse microorganisms may underly more subtle relationships between microbial community structure and ecosystem function.

The microbial production and consumption of nitrous oxide (N_2_O) is a timely area of focus for investigating links between microbial community dynamics and ecosystem processes. Nitrous oxide is currently the third most important greenhouse gas behind CO_2_ and methane and is the predominant ozone-depleting substance emitted in the 21st century^31,32^. Current models estimate that marine ecosystems account for 10 to 53% of the annual global N_2_O emissions, leaving much to be learned about the drivers of spatiotemporal variability^33^. In the marine environment, N_2_O is produced primarily as a by-product of ammonium oxidation or as a free-intermediate in the sequential reduction of nitrate and nitrite to dinitrogen gas (N_2_) during heterotrophic or sulfide-driven denitrification^34^. Elevated rates of production from both pathways are observed near the boundaries of marine oxygen deficient zones (ODZs) and anoxic basins where oxidative and reductive processes are closely coupled in space and time^35–37^. Conversely, the reduction of N_2_O to N_2_ by organisms possessing N_2_O-reductases is the only confirmed biological sink for N_2_O and can drive N_2_O concentrations toward undetectable levels in some anoxic water masses^38,39^. Nitrous oxide cycling is thus the product of distributed metabolic networks that involve syntrophic interactions between important players in the biogeochemical cycling of carbon, nitrogen, and sulfur, with net fluxes likely determined by a complex interplay between environmental and biological factors.

Saanich Inlet is a well-studied, seasonally euxinic fjord located on Vancouver Island, Canada that is characterized by extreme seasonal shifts in water column redox gradients driven by cycles of primary production and physical mixing^40–42^. The inlet is further distinguishable from open ocean ODZs by its restricted depths (~225 m maximum) and the presence of sulfidic bottom water throughout much of the year^43–45^. However, the reliable transition between periods of water column stagnation and bottom water anoxia, and oxygenation of the deep basin following deep water renewal provides a unique opportunity to explore links between biogeochemical rate processes and microbial community dynamics under changing redox conditions^46,47^. Furthermore, studies focusing on microbial community dynamics in easily accessible anoxic basins with reliable sulfide accumulation are useful for understanding trajectories in coastal systems experiencing increases in the frequency of bottom water hypoxia and transient sulfidic conditions^48–50^. Previous work in Saanich Inlet suggests that N_2_O production is driven by ammonium oxidation at oxycline depths, with substantial contributions from reductive processes near the base of the oxycline during periods of bottom-water anoxia and in the deep basin following renewal events^44,51^. However, little is currently known about the role of keystone taxa, and ecological interactions more broadly, in mediating N_2_O-cycling rate processes across marine redox gradients.

This study combines high-throughput sequencing of microbial 16S rRNA amplicons, in situ rate measurements, and environmental characterizations collected in Saanich Inlet over a bi-monthly timeseries between April and October 2018. We leverage network and multivariate statistical analyses to separate co-occurring taxa into discrete subnetworks with contrasting ecological distributions and roles in water column N_2_O cycling. Oxycline subnetworks were correlated with nitrification rates and N_2_O supersaturations and contained keystone taxa implicated in aerobic organic matter remineralization, including members of the ubiquitous SAR11 group. Members of the low-oxygen subnetwork demonstrated a preference for anoxic and N_2_O-undersaturated waters and contained keystone taxa belonging to groups associated anaerobic carbon, nitrogen, and sulfur cycling such as SUP05. Taxa identified as potential keystones belonged to groups found throughout global ODZs and anoxic basins, thus providing a theoretical basis for further investigations into the importance of ecological interactions in regulating marine N_2_O production and accumulation.

## Results

### Microbial community structure

We generated 818,133 paired-end microbial 16S rRNA gene sequence reads across 24 samples. A total of 469,628 bacterial and 211,767 archaeal reads remained following sequence merging and quality filtering, resulting in 168 archaeal and 2814 bacterial non-singleton amplicon sequence variants (ASVs). Mean amplicon lengths were 437 and 445 bp for bacterial and archaeal sequences, respectively. The total number of merged reads for each sample following quality control are reported in Supplementary Table 2.

**Fig. 1:**
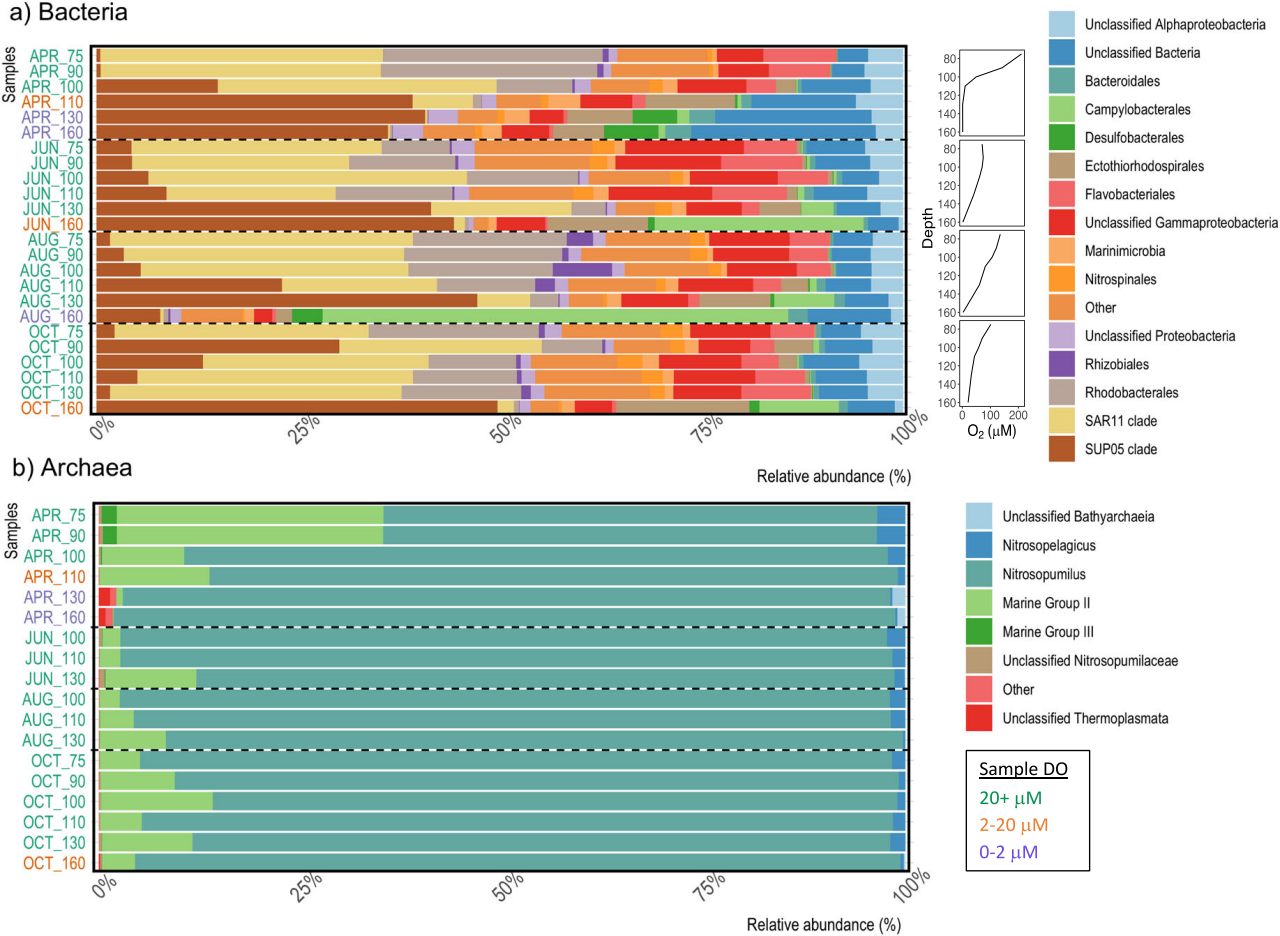
Bacterial and archaeal community structure. Compositional barplots showing relative abundances of **a** Bacteria and **b** Archaea ASVs in Saanich inlet seston samples. Dissolved oxygen profiles for each sampling period are reported in panel (**a**). Samples were obtained from Saanich inlet between April and October 2018. Sample labels on the vertical axes correspond to sampling month and water column depth. Vertical axis labels are color coded according to in situ dissolved oxygen concentrations.

The Saanich Inlet microbial community was well-stratified in April and demonstrated stark shifts in bacterial community structure across depth-dependent redox gradients (Fig. 1a). Members of the SAR11 α-proteobacteria dominated bacterial sequence reads between 75 and 100 m (~35%), along with high abundances of Rhodobacterales (9–27%) and Flavobacteriales (4–9%). These groups decreased in prominence with depth along the oxycline, collectively accounting for <1% of sequence reads below the anoxic interface (130 and 160 m). ASVs belonging to the SUP05 γ-proteobacteria showed contrasting distributions, with maximum values observed in the lower oxycline and anoxic basin between 110 and 160 m (39–41%). Other prominent taxa of the low-oxygen communities included members of the Marinimicrobia (2–4%), Ectothiorhodospirales (6–11%), and Desulfobacterales (0.3–6%). Members of the *Nitrospina* genus within the order Nitrospinales also reached peak relative abundances of 1–1.5% in the lower oxycline and anoxic basin samples despite pronounced vertical stratification of individual ASVs.

Archaeal sequence reads were dominated by Thaumarchaeota ASVs that mapped to two genera within the *Nitrosopumilaceae* family (Fig. 1b). A single *Nitrosopumilus*-like ASV (ARCH1) accounted for 56–86% of all archaeal sequences and was uniformly distributed across water column depths and sampling dates (Supplementary Fig. 2b). Lower oxycline and anoxic basin samples were characterized by increases in the abundance of a second, low-oxygen *Nitrosopumilus* ecotype (ARCH3) with maximum values of over 25% of *Nitrosopumilaceae* reads between 110 and 160 m alongside ASVs belonging to the *Bathyarchaeia* and Thermoplasmata. In contrast, thaumarchaeotal communities of the upper oxycline contained higher abundances of *Nitrosopelagicus*-like variants (Fig. 1, Supplementary Fig. 2b). Members of the Marine Group II (MGII) and Marine Group III (MGIII) Euryarchaeota also reached peak relative abundances in upper oxycline samples (~33% and 2%, respectively), and decreased precipitously with depth (Fig. 1b).

At least three discrete renewal events were detected at variable depths prior to sampling in June, August, and October, resulting in substantial changes to water column redox gradients and microbial community structures across renewal depths^51^. Renewal events prior to June and August sampling impacted midwater depths between 75 and 150 m, while October renewal was associated with oxygenation of the deep basin below 160 m. Increases in dissolved O_2_, NO_3_^−^, and N_2_O concentrations across renewal depths were associated with elevated abundances of SAR11, *Rhodobacteraceae* and *Flavobacteraceae* ASVs in addition to vertical homogenization of the Archaeal community (Fig. 1). These events were generally accompanied by decreases in the relative abundance of SUP05, Marinimicrobia, Ectothiorhodospirales, and Desulfobacterales ASVs at deeper renewal depths and upward transport to 75 and 90 m ostensibly resulting from uplift of anoxic basin waters and subsequent mixing with renewal waters. Progressive increases in the prevalence of ASVs belonging the Campylobacterales were also detected at 130 and 160 m between April and August. Campylobacterales reads were dominated by a single *Arcobacter* ASV that accounted for between 25% and 57% of total bacterial reads at 160 m in June and August, respectively. Conversely, *Nitrospina* ASVs increased in relative abundance with time throughout much of the water column, with peak values of approximately 3% occurring at mid-depth in October despite variable depth-related trends between sampling dates.

**Fig. 2:**
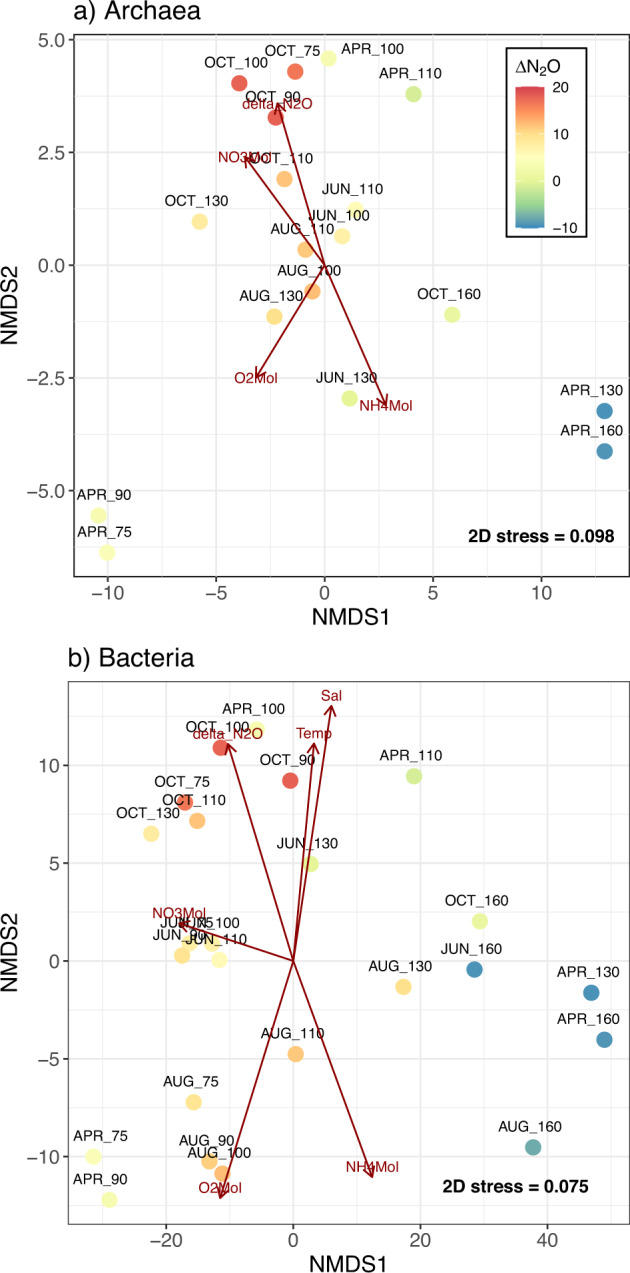
Environmental drivers of community dissimilarity. Nonmetric multidimensional scaling (NMDS) ordinations for **a** Archaea and **b** Bacteria communities. NMDS analyses were conducted using Aitchison distances between samples calculated using clr-transformed ASV tables. Significant environmental predictors of community dissimilarity were calculated using *envfit* and the corresponding vectors are represented by red arrows.

Clustering of bacterial and archaeal communities via non-metric multidimensional scaling (NMDS) followed water column N_2_O saturations (ΔN_2_O), with samples from undersaturated waters grouping together closely (Fig. 2). Envfit analysis implicated NO_3_^−^ concentrations as the strongest predictor of community structure for both bacterial (*r*^2^ = 0.82, *p* = 0.001) and archaeal (*r*^2^ = 0.77, *p* = 0.001) ASVs. Secondary predictors for both domains included dissolved O_2_ concentrations, NH_4_^+^ concentrations and ΔN_2_O. The influence of dissolved O_2_ on NMDS ordinations was stronger for bacterial communities (*r*^2^ = 0.70, *p* = 0.001) than for archaeal communities (*r*^2^ = 0.65, *p* = 0.001), while ΔN_2_O showed greater influence over archaeal community structure (*r*^2^ = 0.65, *p* = 0.001 for Archaea versus *r*^2^ = 0.57, *p* = 0.001 for Bacteria). In contrast, the influence of NH_4_^+^ concentration was similar between the bacterial (*r*^2^ = 0.69, *p* = 0.001) and archaeal domains (*r*^2^ = 0.71, *p* = 0.002). Temperature and salinity were also implicated as potential drivers of community structure, although correlational strengths were lower in comparison to other variables (*r*^2^ = 0.47–0.52, *p* = 0.001–0.015).

### N_2_O-cycling community networks

**Fig. 3:**
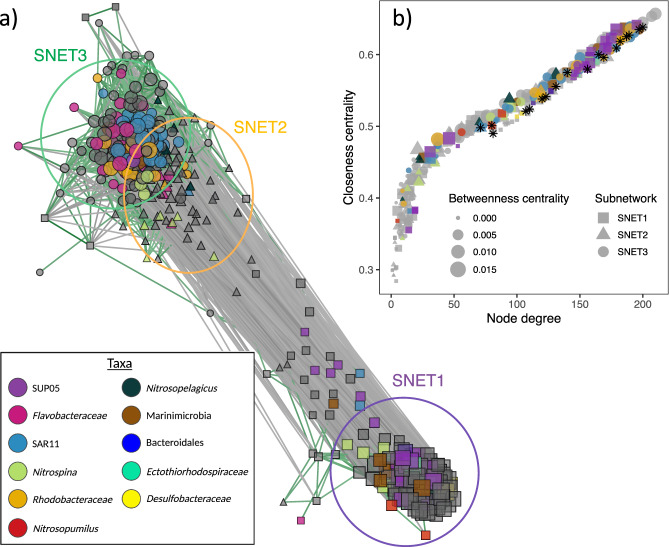
Co-occurrence network depicting interactions between bacterial and archaeal ASVs in the Saanich Inlet microbiome. In panel **a** gray lines depict negative covariance and green lines depict positive covariance with ρ > |0.60|. Node size represents intranetwork connectivity (K_in_) and shapes indicate subnetwork assignments determined by WGCNA. Squares, triangles, and circles represent taxa belonging to SNET1, SNET2, and SNET3, respectively, and are highlighted by colored circles. Taxonomic assignments are represented by colored nodes. Panel **b** depicts relationships between node (ASV) degree, closeness centrality, and betweenness centrality determined through analysis of the propr network. Individual ASVs implicated by sPLSR (Fig. 5) as important predictors of rate processes and ΔN_2_O are indicated (*) in panel (**b**).

We performed two separate network-level analyses on the combined, centred-log ratio (clr) transformed bacterial and archaeal ASV tables to explore patterns of community assembly and relationships between community interaction networks and N_2_O cycling. A total of 38 archaeal and 324 bacterial ASVs were included in the network-level analyses following removal of low abundance taxa to improve interpretability and minimize the risk of spurious correlations. First, we used proportionality analyses to define a community co-occurrence network of interacting taxa with absolute rho values >0.60 (Fig. 3). These results were then compared with those obtained through weighted gene correlational network analysis (WGCNA) of the same dataset to identify core community members and elucidate links between microbial community structure and N_2_O production processes. Relationships between entire community subnetworks and N_2_O production were assessed by correlating subnetwork eigengenes with relevant sample traits (environmental variables and measured rates). The potential role of community structure in mediating water column N_2_O-cycling was explored by evaluating relationships between ASV subnetwork membership, intranetwork connectivity (K_in_), and ASV importance in predicting rate processes and water column N_2_O saturations (ΔN_2_O).

**Fig. 4:**
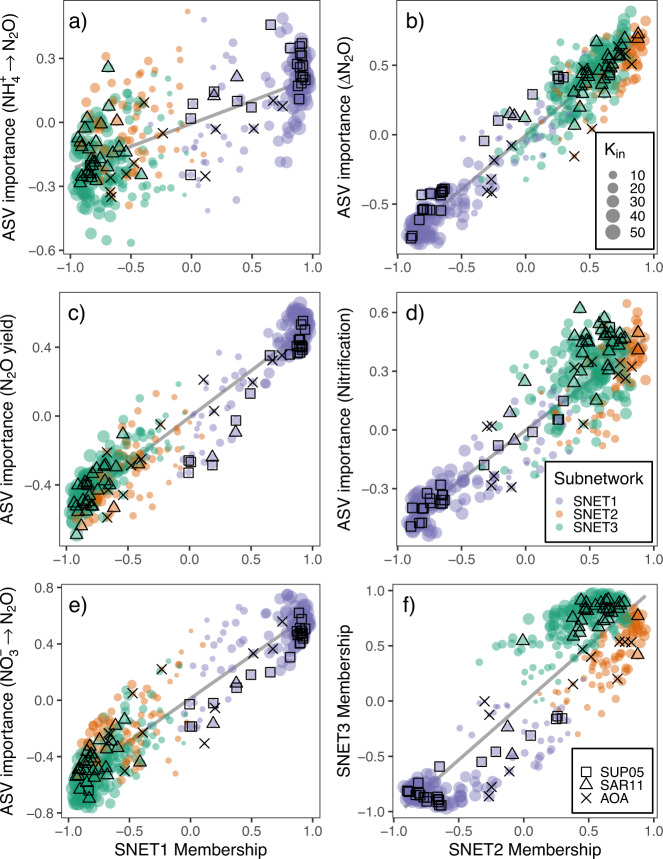
Relationships between ASV subnetwork memberships and ASV importance in predicting various N_2_O cycle proxies. Left panels depict relationships between SNET1 memberships and ASV importance in predicting **a** rates of N_2_O production from NH_4_^+^ oxidation, **c** N_2_O yields from nitrification, and **e** rates of N_2_O production from NO_3_^−^ reduction. Right panels depict relationships between SNET2 memberships and **b** ΔN_2_O, **d** nitrification rates, and **f** SNET3 memberships. ASV importance corresponds to the Pearson coefficients calculated for pairwise correlations between microbial ASVs and sample traits. Colors correspond to community subnetworks inferred from WGNA and bubble size indicates degree of intranetwork connectivity (K_in_) for each ASV. Black squares, crosses, and triangles denote ASVs belonging to the SUP05 clade, the Nitrosopumilaceae family, and SAR11 clade, respectively.

WGCNA clustered microbial ASVs into three discrete subnetworks (SNETs 1–3) containing 135, 77, and 150 ASVs, respectively (Table 1). These results were largely congruent with those identified through proportionality analyses, with taxa separating broadly into three primary clusters according to their subnetwork assignments identified through WGCNA (Fig. 3). SNET1 was inversely correlated with dissolved O_2_ (*r* = −0.75, *p* = 3 × 10^−4^), ΔN_2_O (*r* = −0.65, *p* = 0.004), and [NO_3_^−^ + NO_2_^−^] (*r* = −0.89, *p* = 9 × 10^−7^), and positively correlated with N_2_O production from NO_3_^−^ reduction (*r* = 0.61, *p* = 0.007) as well as N_2_O yields from nitrification (*r* = 0.55, *p* = 0.02) (Fig. 4, Table 1). A total of 81 SNET1 ASVs demonstrated significant positive correlations to rates of N_2_O production from NO_3_^−^ reduction (*r* = 0.47–0.75, *p* < 0.05), while 52 ASVs correlated significantly with N_2_O yields from nitrification (*r* = 0.47–65, *p* < 0.05) (Supplementary Data 1). ASV membership to SNET1 was strongly correlated to ASV importance in predicting both N_2_O production rates and nitrification yields, and inversely correlated with ΔN_2_O (Fig. 4). Although SNET1 membership was also positively correlated with ASV importance in predicting rates of N_2_O production from NH_4_^+^ production, taxa-specific correlations were generally weak and insignificant (Fig. 4, Supplementary Data 1).

**Table 1:** Pairwise correlations between subnetwork (SNET) eigengenes and sample traits.

Environmental variables						Rate processes			
SNET	# of taxa	O_2_	ΔN_2_O	NO_3_^−^ + NO_2_^−^	NH_4_^+^	Nitrification	NH_4_^+^ → N_2_O	NO_3_^−^ → N_2_O	N_2_O yield
1	135	**−0.75**	**−0.65**	**−0.89**	**0.72**	−0.42	0.21	**0.61**	**0.55**
		**(3e−04)**	**(0.004)**	**(9e−07)**	**(8e−04)**	(0.08)	(0.40)	**(0.007)**	**(0.02)**
2	77	0.36	**0.71**	**0.74**	**−0.73**	0.44	−0.078	−0.32	**−0.49**
		(0.10)	**(0.001)**	**(4e−04)**	**(6e−04)**	(0.07)	(0.80)	(0.20)	**(0.04)**
3	150	**0.83**	**0.52**	**0.83**	**−0.63**	0.38	−0.25	**−0.64**	**−0.49**
		**(2e−05)**	**(0.03)**	**(2e−05)**	**(0.005)**	(0.10)	(0.30)	**(0.004)**	**(0.04)**

Relationships are reported as Pearson’s correlation coefficients with corresponding *p*-values (in parentheses). N_2_O production rates from NH_4_^+^ oxidation and NO_3_^−^ reduction are symbolized by black arrows.

Statistically significant correlations (*p* < 0.05) are bolded.

SNET2 and SNET3 represented nested subnetworks that displayed overlapping niche distributions and similar relationships to environmental parameters and process rates (Fig. 4, Table 1). SNET2 was most strongly correlated to ΔN_2_O (*r* = 0.71, *p* = 0.001) and [NO_3_^−^ + NO_2_^−^] (*r* = 0.75, *p* = 3 × 10^−4^), but was not significantly related to dissolved O_2_ concentrations. In contrast, SNET3 demonstrated significant positive correlations to dissolved O_2_ (*r* = 0.83, *P* = 2 × 10^−5^), [NO_3_^−^ + NO_2_^−^] (*r* = 0.83, *p* = 2 × 10^−5^), and ΔN_2_O (*r* = 0.52, *p* = 0.03). Positive correlations observed between SNET2 and SNET3 and nitrification rates were not statistically significant at the subnetwork level (*r* = 0.44 and 0.38, *p* = 0.07 and 0.10, respectively) (Table 1). Regardless, we detected 25 bacterial ASVs with significant associations to nitrification rates (*r* = 0.47–0.65, *p* < 0.05) and a strong positive correlation between ASV importance in predicting nitrification rates and ASV membership in SNET2 and SNET3 (Fig. 4, Supplementary Data 1). ASV membership in SNET2 and SNET3 was also strongly associated with water column ΔN_2_O values, with a total of 42 ASVs demonstrating strong positive correlations (*r* = 0.60–0.89, *p* < 0.001).

### Keystone taxa linked to N_2_O production processes

We extended our network inferences further by utilizing network topological indices of the propr covariance network to identify putative keystone taxa amongst core community members that correlated strongly with rate processes and water column N_2_O saturations (ΔN_2_O)^52^. We considered high node (ASV) degree, closeness centrality, and betweenness centrality measures as indicators of potential keystone status for microbial ASVs^28,53,54^.

Core community taxa in SNET1 that correlated significantly with rates of N_2_O production from NO_3_^−^ reduction included highly connected members of the *Desulfobacteraceae*, *Ectothiorhodospiraceae*, Bacteroidales, and the SUP05 clade (Fig. 3, Supplementary Data 1). Other prominent ASVs related to N_2_O production from NO_3_^−^ reduction in SNET1 included members of the *Nitrosopumilus* and *Nitrospina* genera (*r* = 0.51–0.59, *p* < 0.027), as well as several unclassified members the α- and γ-proteobacteria, and Marinimicrobia. Core community members that correlated significantly with elevated nitrification rates and ΔN_2_O belonged primarily to the SAR11 clade (11 of 25 ASVs) in addition to variants from the *flavobacteraceae* and MG II Euryarchaeota, which were generally well connected in SNET2 and SNET3 (Fig. 3, Supplementary Data 1). Additional taxa with high levels of connectivity in SNET2 and SNET3 that correlated strongly with ΔN_2_O and demonstrated positive associations with nitrification rates included four *Nitrosopelagicus*-like variants, and several *Rhodobacteraceae* and *Verrucomicrobiae* ASVs.

**Fig. 5:**
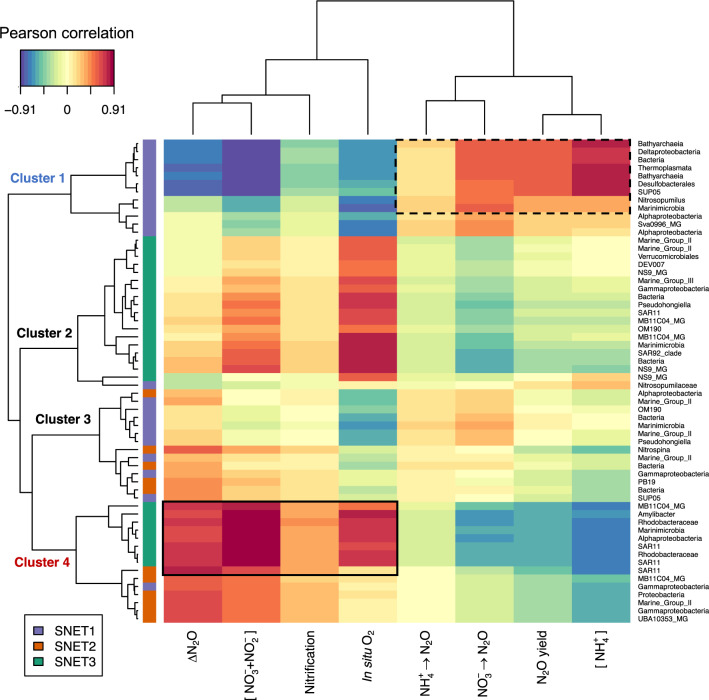
Relationships between prokaryotic amplicon sequence variants (ASVs), relevant environmental variables, and process rates. N_2_O production rates from NH_4_^+^ oxidation and NO_3_^−^ reduction are symbolized by black arrows. Pairwise correlation coefficients between ASVs and sample traits were calculated using a two-component sPLS regression model and are presented as a clustered heatmap. Taxa with correlations to nitrification rates >0.30 are indicated by solid black lines and taxa with correlations to N_2_O production from NO_3_^−^ reduction >0.50 are indicated by dashed lines. Hierarchical clustering of variables was achieved using a complete Euclidean distance method. ASV subnetwork assignments determined through WGCNA are indicated by colored rectangles on the vertical axis dendrogram. Taxonomic labels correspond to the lowest level of classification determined for each ASV through alignment with the SILVA database.

Microbial taxa that correlated well with N_2_O production rates, nitrification rates, and ΔN_2_O generally scored high on network topological indices of keystone status (Fig. 3b). However, several highly connected ASVs suggested by WGCNA as potentially important with respect to N_2_O cycling were not classifiable below the class level and many were classifiable only at the kingdom level (Supplementary Data 1). Furthermore, results of the WGCNA demonstrating correlations between individual ASVs and rate processes are difficult to interpret given the large number of taxa implicated. In attempt to circumvent this issue, we fit a sparse partial least squares regression (sPLSR) model to predict sample traits from clr-transformed microbial ASV tables. sPLSR allowed us to elucidate robust taxa-specific links between individual ASVs and N_2_O production processes by introducing a LASSO penalization to remove taxa with negligible effects^55^. The final model was built using two latent components and a total of 60 microbial ASVs that separated broadly into four primary clusters (Fig. 5).

Cluster 1 contained eight bacterial and four archaeal ASVs, all of which belonged to SNET1 and correlated positively with N_2_O production from NO_3_^−^ reduction (*r* = 0.38–0.60) (Supplementary Data 2). The strongest taxon-specific correlations to rates of N_2_O production from NO_3_^−^ reduction (*r* > 0.50) were observed for ASVs that also correlated well with N_2_O yields from nitrification (*r* = 0.38–0.60), which included *Nitrosopumilus* and SUP05 variants in addition to members of the Desulfobacterales, Marinimicrobia, Bathyarchaeia, and Thermosplasmata. In contrast, a subset of nine bacterial taxa within Cluster 4 demonstrated strong correlations to ΔN_2_O (*r* = 0.62–0.78) and moderate correlations to nitrification rates >0.30 (Fig. 5). Taxa in this subcluster were also positively associated with dissolved O_2_ and NO_3_^−^ + NO_2_^−^ concentrations. ASVs included three SAR11 variants, three *Rhodobacteraceae* variants (including one *Amylibacter* ASV), one member of the *Puniceicoccaceae* (Verrucomicrobia MB11C04 Marine Group), and unclassified members of the α-proteobacteria and Marinimicrobia. Cross-referencing these results with those of our network analyses shows that individual ASVs implicated by sPLSR as strongest predictors of N_2_O cycling rate were also implicated by our network analysis as potential keystone taxa with high closeness centrality and node degree (Fig. 3b, Supplementary Fig. 3).

## Discussion

Spatiotemporal trends in microbial community structure were largely consistent with literature surveys of the Saanich Inlet water column over the seasonal stratification cycle^46,56^. Vertical stratification of key microbial taxonomic groups was also in agreement with patterns of redox-driven niche partitioning observed in open ocean ODZs and other coastal anoxic basins^10,11^. Given the extensive body of existing literature documenting patterns of microbial community composition across water column redox gradients and the well-characterized seasonal succession patterns observed in Saanich Inlet, we direct subsequent discussion toward key microbial players and community interaction networks implicated in N_2_O cycling.

The Saanich Inlet archaeal community was dominated by thaumarchaeotal ASVs belonging to the *Nitrosopumilaceae* family, a highly supported monophyletic clade that contains all known members of the ammonium oxidizing archaea (AOA)^57^. The majority of putative AOA sequences belonged to a single *Nitrosopumilus*-like ASV, which displayed broad water column distributions throughout the sampling period. This variant clustered together in SNET1 alongside two additional *Nitrosopumilus*-like ecotypes that were enriched in samples from the lower oxycline and anoxic basin. Members of the *Nitrosopumilus*-like ecotype are widely distributed across marine ecosystems, suggesting a role as generalists possessing broad environmental tolerances^58–60^. *Nitrosopumilus*-like variants have been reported to dominate AOA communities in oxygen-depleted waters of other sulfidic basins, including the Baltic and Black Seas, and in ODZ waters of the Eastern Tropical South Pacific^9,61,62^. Recent work has demonstrated oxygen production by *Nitrosopumilus maritimus* cultures as a means of supporting ammonium oxidation under anoxic conditions, indicating the presence of unique cellular machinery for maintenance during periods of severe oxygen limitation^63^. In contrast, putative AOA variants related to the *Nitrosopelagicus* genus, which belong to the previously delineated water column group A (WCA) clade^64,65^, clustered together in SNET2 and were most prevalent at oxycline depths. Previous surveys of oxygen-deficient water columns have also demonstrated a preference of WCA-type AOA for oxygenated, epipelagic waters, suggesting that low-oxygen adaptation may not be universally distributed across all AOA clades^62,66,67^.

Inferences based on experimentally derived rate measurements used in this study and relationships between ΔN_2_O and apparent oxygen utilization indicate that N_2_O production in Saanich Inlet is dominated by ammonium oxidation across oxycline depths^44,51^. Similar to previous work in Saanich Inlet, we did not detect any sequences related to known ammonium oxidizing bacteria, suggesting that ammonium oxidation is predominantly mediated by AOA^7^. However, associations between biological variables and rates of N_2_O production from ammonium oxidation were generally weak despite a significant correlation between the low-oxygen subnetwork (SNET1) and nitrification N_2_O yields. Consistent with previous work, a substantial drop-off in overall nitrification rates was observed at O_2_ concentrations less than 1 μmol L^−1^ resulting in low overall N_2_O production rates despite extremely high molar N_2_O yields (Fig. 4a)^51,68^. In contrast, maximum rates of N_2_O production from NH_4_^+^ oxidation were measured at 160 m in October under suboxic conditions (O_2_ < 20 μmol L^−1^) following renewal of the deep basin^51^ (Fig. 4a). Injection of oxygen to the deep basin combined with high NH_4_^+^ concentrations in this case appears to have stimulated nitrification and the associated production of N_2_O at relatively high yields.

Closer analysis of the data following removal of the October outlier point revealed a linear dependence of N_2_O production rates from ammonium oxidation on overall nitrification rates (Fig. 4b). Furthermore, nitrification end products (NO_3_^−^ + NO_2_^−^) and N_2_O supersaturations were strongly associated with SNET2 and SNET3 communities (oxycline subnetworks hereafter), which contained distinct *Nitrosopelagicus*-like and *Nitrosopumilus*-like ecotypes. Water column N_2_O accumulation in Saanich Inlet thus appears driven primarily by *Nitrosopumilus-* and *Nitrosopelagicus*-like AOA variants at low to moderate yields across oxycline depths, with low-O_2_ *Nitrosopumilus*-like ecotypes dominating the high-yield production of N_2_O near the anoxic boundary and in the deep basin following oxygen renewal. However, putative nitrifying taxa (AOA or NOB) were not implicated by sPLSR or WGCNA as significant predictors of nitrification rates. We also did not detect any systematic variation in nitrification rates associated with dissolved O_2_ or NH_4_^+^ concentrations (Supplementary Fig. 5), suggesting that alternative mechanisms may be responsible for regulating variability in nitrification rates, and thus N_2_O accumulation across oxycline depths.

Consideration of community-wide dynamics showed that core community members belonging to oxycline subnetworks demonstrated stronger relationships to both nitrification rates and ΔN_2_O. Putative keystone taxa linked to nitrification rates belonged primarily to groups previously affiliated with the heterotrophic remineralization of organic matter, including the SAR11 α-proteobacteria, *Rhodobacteraceae, Flavobacteriaceae*, and MGII Euryarchaeota. These results were largely substantiated by the sPLSR analysis, which implicated prominent members of the SAR11 clade, *Rhodobacteraceae*, and *Puniceicoccaceae* (Verrucomicrobia) as important predictors of ΔN_2_O and nitrification rates. Members of the SAR11 clade are found ubiquitously throughout the global ocean and across ODZ redox boundaries, and generally possess streamlined genomes adapted for aerobic growth on dissolved organic carbon under oligotrophic conditions^69,70^. Conversely, groups affiliated with the Verrucomicrobia, *Rhodobacteraceae*, *Flavobacteriaceae*, and MGII Euryarchaeota are commonly observed in association with phytoplankton blooms and may act as specialist consumers of various phytoplankton-derived carbon substrates^71–74^. The presence of proteorhodopsins in MGII metagenomes also suggests the potential for photoheterotrophic growth within the euphotic zone for this particular group^75^.

Interestingly, taxa affiliated with many of these groups have been identified as keystone community members across a wide range of aquatic systems, further highlighting the importance of common aerobic heterotrophs in maintaining community stability and facilitating ecosystem function^28^. For example, *Rhodobacteraceae* groups can form mutualistic cross-feeding relationships with pelagic diatoms whereby remineralized ammonium stimulates carbon fixation^76^. Similarly, co-culture experiments using *Synechococcus* and *Roseobacter* populations have demonstrated that interactions between marine phototrophs and heterotrophs are stabilized over time through mutualistic nutrient cycling that involves leakage and subsequent remineralization of organic matter^77^. Recent work has indicated that marine AOA also exude considerable amounts of labile dissolved organic matter, and that these exudates may support the growth requirements of auxotrophic heterotrophs such as SAR11^78^. Indeed, regeneration of ammonium or urea from dissolved organic matter has been proposed as a potential mode of metabolic coupling between marine AOA and SAR11 based on metaproteomic surveys of the Saanich Inlet water column^7^. This is consistent with previous studies that have linked ammonium and nitrite oxidation rates to organic matter export and remineralization in marine water columns^79,80^. These results collectively suggest that nitrification may be modulated through cross-feeding interactions with common aerobic heterotrophs occupying diverse niche spaces across oxycline depths.

Appreciable rates of N_2_O production from NO_3_^−^ reduction (hereafter denitrification), on the other hand, were primarily restricted to suboxic and anoxic depths, and were associated with putative keystone taxa belonging to the low-oxygen subnetwork. Prominent SUP05 ASVs were highly connected within the low-oxygen subnetwork and demonstrated robust taxa-specific correlations to N_2_O production rates in both the WGCNA and sPLSR analyses. This is consistent with the role of SUP05 as core community taxa linking the biogeochemical cycling of nitrogen and sulfur in oxygen-depleted environments^7,9,81^. Members of the SUP05 clade are abundant and active members of microbial communities across marine redox boundaries even in the absence of detectable levels of hydrogen sulfide and have been implicated as important drivers of autotropic denitrification coupled to sulfide and elemental sulfur oxidation^82–84^. Several mechanisms have been proposed to explain the persistence of SUP05 in sulfide-free waters, including intracellular storage of elemental sulfur or particle-associated micro-niches^84–86^. Metagenomic and metaproteomic surveys of marine ODZs and coastal anoxic basins also indicate that the majority of SUP05 variants lack the metabolic machinery required to reduce N_2_O to dinitrogen gas (N_2_), leading to further speculation about a potential role in water column N_2_O production^7,81,87^. Given that SUP05 also appear as the only organisms in Saanich Inlet to express consecutive denitrification genes, our results provide further evidence that SUP05 acts as an important mediator of N_2_O production from denitrification^7^.

Several additional ASVs were also implicated by our analyses as potential keystone taxa with significant links to N_2_O production from denitrification, including members of the Marinimicrobia, Bathyarchaeia, *Ectothiorhodospiraceae*, Desulfobacterales, and Thermoplasmata. Whether these relationships reflect direct contributions to denitrification processes, ecological interactions with denitrifying taxa, or overlapping niche-preferences remains to be determined. Genes encoding components of the denitrification pathway are spread ubiquitously throughout the prokaryote domains with many taxa possessing only a partial compliment of those required for complete denitrification, making it difficult to identify putative functional groups based on 16S rRNA sequences alone^4^. Furthermore, a considerable proportion of highly connected ASVs within the low-oxygen subnetwork were not classifiable below the phylum or class level, leaving much to be learned about the taxonomic affiliations and functional potentials of many potential core community members. Approximately half of the total denitrification proteins detected in Saanich Inlet belong to taxa other than SUP05^7^, indicating potential contributions to N_2_O production facilitated by extracellular exchange of metabolic intermediates between modular components of the denitrification pathway. For example, we detected two low-oxygen SAR11 ecotypes, and recent analysis of single-cell genomes from the ETNP has uncovered novel ODZ variants that contribute to nitrogen loss processes via respiratory nitrate reduction^70^.

As noted previously, elevated rates of N_2_O production from denitrification were observed at depths depleted of nitrate and nitrite and were likely augmented by substrate enrichment following tracer additions^51^. However, putative nitrite-oxidizing bacteria (NOB) from the *Nitrospina* genus consisted of several low-O_2_ ecotypes that were well-connected in the low-oxygen subnetwork alongside low-oxygen AOA ecotypes and correlated significantly with rates of N_2_O production from denitrification. This correlation with denitrification supports previous reports of ecotype-specific metabolic interactions between AOA and NOB across depth-dependent environmental gradients and suggests the potential for N_2_O production at lower oxycline depths driven by coupled nitrification-denitrification^88–90^. Surveys of the Eastern Tropical North Pacific (ETNP) reported enrichment of novel *Nitrospina*-like variants at the upper ODZ boundary and within the ODZ core coinciding with relatively high rates of nitrite oxidation coupled to nitrate reduction^91,92^. Measurable rates of nitrification were also detected at sub-micromolar oxygen concentrations during our sampling period^51^, and active ammonium and nitrite oxidation has been demonstrated in ODZ waters at oxygen concentrations as low as 5 nmol L^−1^ ^51,68^. Regardless, taxa belonging to the low-oxygen subnetwork generally displayed strong negative correlations with water column ΔN_2_O, and appreciable rates of N_2_O production from denitrification were generally concomitant with pronounced N_2_O undersaturation^51^. As a result, N_2_O production near the anoxic interface appears off-set by close coupling with N_2_O consumption processes. Tight metabolic coupling between distributed elements of the denitrification pathway in Saanich Inlet may explain the low water column N_2_O concentrations and surface N_2_O fluxes relative to open ocean ODZs^44^.

**Fig. 6:**
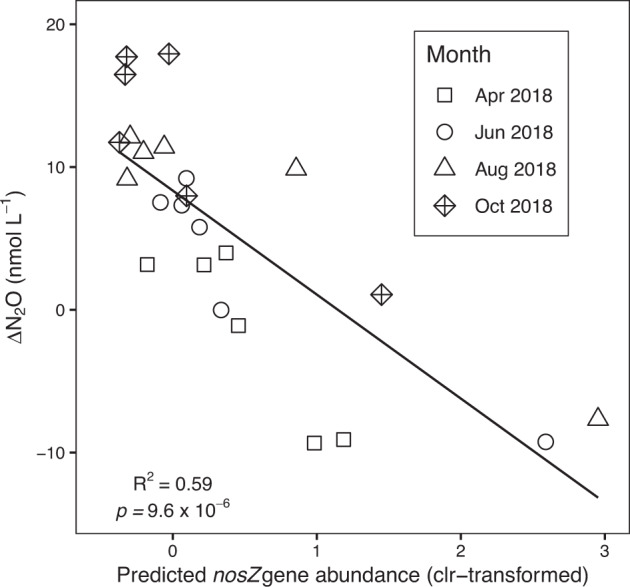
Relationship between water column ΔN_2_O (nmol L^−1^) and PICRUSt2-predicted bacterial *nosZ* gene abundances. *NosZ* gene abundances were inferred from the predicted KEGG orthologues across all 24 samples using bacterial 16S rRNA amplicon sequences as input to the PICRUSt2 algorithm.

Several of the core community members identified in the low-oxygen subnetwork belong to taxonomic groups containing ODZ representatives that possess atypical N_2_O reductases, such as the *Ectothiorhodospiraceae, Arcobacteraceae*, and Bacteroidales^12,93^. PICRUSt2 predictions of bacterial *nosZ* gene abundances based on 16S rRNA sequences were negatively correlated with ΔN_2_O (Fig. 6), supporting previous reports of elevated *nosZ* activity within the Saanich Inlet deep basin^7^. Organisms possessing the atypical *nosZ* variant are commonly associated with higher N_2_O affinities and lower O_2_ sensitivities, and also typically lack additional genes in the denitrification pathway^93–95^. A conceptual model describing mutualistic N_2_O-cycling interactions has already been proposed in which N_2_O produced by SUP05 is used by *nosZ*-harboring Marinimicrobia ecotypes to store polysulfide and regenerate H_2_S^12^. In contrast, atypical *nosZ* in the Bacteroidia class has been linked to particle-associated N_2_O consumption while members of the *Arcobacter* genus in Saanich Inlet have been implicated in sulfide-driven denitrification^93,96,97^. Atypical *nosZ* genes have also been identified in members of the *Ectothiorhodospiraceae*, a group generally associated with sulfide oxidation in anoxic environments^93,98,99^. Given the inability of SUP05 in Saanich Inlet to reduce N_2_O, these results indicate that N_2_O consumption may be mediated through cross-feeding relationships involving diverse N_2_O-reducing organisms that occupy varying ecological niches^7,8^.

The results of our analyses provide statistical support for the presence of distributed metabolic networks mediating N_2_O production and consumption in low-oxygen and sulfidic environments, and implicate additional groups involved in anaerobic sulfur cycling as potential keystone taxa. ASVs belonging to the *Desulfobacteraceae* family, for example, may supply sulfide to denitrifying organisms by coupling heterotrophic carbon oxidation to sulfate reduction^83,100^. Metabolic coupling between chemolithotrophic denitrifiers and heterotrophic sulfate reducers may also help to explain the presence of detectable N_2_O production rates from denitrification observed under well-oxygenated conditions following deep water renewal^51^. Appreciable rates of N_2_O production from reductive processes were detected at oxycline depths with O_2_ concentrations as high as 70 μmol L^−1^ following summer and fall renewal events and were concomitant with high relative abundances of taxa from the low-oxygen subnetwork resulting from uplift of anoxic basin waters. Taxa from the low-oxygen subnetwork thus appear to serve as a net N_2_O sink during periods of stable water column stratification yet maintain the capacity to respond rapidly to fresh inputs of terminal electron acceptors following renewal events, even under aerobic conditions. Re-supply of oxygen and fixed nitrogen species following deep-water renewal events therefore has the potential to simultaneously stimulate rates of N_2_O production from all pathways and impede N_2_O reduction within the deep basin, and may contribute to the elevated surface N_2_O fluxes typically observed over fall months^44^.

It is important to reiterate that specific findings obtained in a dynamic anoxic fjord that experiences both sulfide accumulation and transient oxygenation of the deep basin may not be extrapolatable to permanent open ocean ODZs. However, many of the interactions reported herein are centered around microbial constituents found ubiquitously throughout other sulfidic basins and open ocean ODZs, suggesting that similar relationships may be important determinants of microbial rate processes in other oxygen-deficient marine systems. Regardless, interpreting the underlying nature of specific co-occurrences revealed by network analyses is challenging, as interactions between taxa may reflect several ecological, environmental, or stochastic mechanisms. This is complicated further by the functional ambiguities associated with molecular marker profiling of natural microbial communities. Although improvements to the taxonomic resolution provided by shotgun metagenomics surveys may permit a more detailed assessment of microbial community interaction networks from a functional perspective, this would not eliminate the need to verify presumed ecological interactions empirically. A comprehensive view of how ecosystem function emerges from the cumulative influences of environmental variability and microbial community dynamics will require careful experimentation guided by exploratory analyses to better understand the mechanisms that drive ecological interactions and co-evolution between microbial taxa over space and time.

## Methods

### Field sampling

Sampling was conducted bimonthly on the *MSV John Strickland* at a single location in Saanich Inlet (48° 37.53’N, 123° 29.91’W) on 5 April, 14 June, 02 August, and 25 October 2018 (Supplementary Fig. 1). The specifics of the sampling campaign, including chemical analyses and ^15^N-labeled tracer experiments to measure N_2_O production rates from NH_4_^+^ oxidation and NO_3_^−^ reduction, have been detailed in Ji et al.^51^. Environmental proxies for N_2_O production processes considered in the statistical analyses included dissolved O_2_, N_2_O saturation, as well as NO_3_^−^ + NO_2_^−^ and NH_4_^+^ concentrations. N_2_O saturation was defined as the N_2_O excess, which is calculated from the concentrations difference between measured and expected equilibrium values with respect to the atmosphere. Seston samples for DNA sequencing were also obtained from the six discrete sampling depths (75, 90, 100, 110, 130, and 160 m) by filtering 5 L of seawater onto 0.2 µm Sterivex™ filters (Merck) by peristalsis. Samples designated for DNA extraction were immediately placed on dry ice, transferred to a −80 °C freezer later the same day, and stored for 6–12 months prior to extraction.

### DNA extractions and high-throughput sequencing

Nucleic acids were extracted according to Crump et al.^101^ with the modifications suggested by Huber et al.^102^ and Sogin et al.^103^. Sterivex filters were thawed, cut into strips, and placed in clean 2 mL microcentrifuge tubes containing 1 mL of DNA extraction buffer (1.5 M NaCl, 0.1 M Na-EDTA [pH 8.0], 0.1 M Tris-HCl [pH 8.0], 0.1 M NaH_2_PO_4_ [pH 8.0], and 5% cetyltrimethylammonium bromide; 0.2 μm filtered, autoclaved). Each tube was aliquoted with 20 μl of Proteinase K (10 mg/ml) and 40 μl of lysozyme (50 mg/ml) and then taken through three freeze-thaw cycles of 15 min at −80 °C and 5 min at 37 °C. Following the final freeze step, tubes were incubated at 37 °C for 30 min prior to addition of 50 μl sodium dodecyl sulfate (SDS; 20%; 0.2 μm filtered and autoclaved) and incubation in a water bath at 65 °C for 120 min. Tubes were then filled with phenol:chloroform:isoamyl alcohol (P:C:I; 25:24:1) to a final volume of ~2 ml, vortexed, and centrifuged (3000 rpm) for 5 min. The aqueous layer was transferred to a new 2 ml tube and the P:C:I addition, spin-down, and transfer steps were repeated a second time. DNA was precipitated by adding 0.6 volumes of molecular grade isopropopanol (99.5%), mixing gently, and incubating at room temperature for 2 h. Samples were then centrifuged (13,000 rpm) for 30 min, washed with 1 ml of ethanol (70%), dried, and eluted in 150 μl of TE buffer (10 mM Tris-HCl [pH 8.0]; 1 mM Na-EDTA [pH 8.0]; 0.2 μl filtered, autoclaved). A sterile, blank Sterivex filter was included in each round of extractions, and the resulting material was carried through the PCR validation steps to ensure no contaminants were introduced during the extraction process. DNA extracts were cleaned using a QIAquick® PCR purification kit and DNA concentrations in cleaned extracts were quantified on a NanoDrop™ One Microvolume UV-Vis Spectrophotometer (Thermo Scientific).

The presence of target genes (16S rRNA) was verified by polymerase chain reaction (PCR) of genomic DNA (gDNA) using primers targeting the bacterial V6-V8 variable regions described by Comeau et al.^104^ (Supplementary Table 1). PCR was conducted in 20 μl reaction volumes containing 4.0 μl 5X Green GoTaq™ reaction buffer (Promega), 2.0 μl dNTPs mixture diluted to final concentrations of 2.0 mM each (Thermo Scientific), 1.0 μl each of 2.0 μM forward and reverse primer (Eurofins Scientific), 10.8 μl UltraPure™ DNase/RNase-Free water (Invitrogen), 0.2 μl GoTaq™ DNA polymerase (Promega) and 1.0 μl template DNA. Thermal cycling began with an initial denaturation at 94 °C for 120 s, followed by 30 cycles of denaturation at 94 °C for 30 s, annealing at 55 °C for 45 s, extension at 72 °C for 120 s, and terminated following a final extension at 72 °C for 600 s. Bacterial and archaeal 16S rRNA genes were selected for sequencing from raw extracts using the same universal primer sets on an Illumina MiSeq at the Integrated Microbiome Resource (Dalhousie University, Halifax, Canada) using 2 × 300 bp paired-end V3 chemistry (https://imr.bio/protocols.html)^104^. Final amplicon read lengths were 437 and 445 bp for Bacteria and Archaea, respectively. Bacterial 16S rRNA gene sequences were obtained from all samples, while archaeal sequences were obtained in 18 of 24 samples.

Demultiplexed reads were trimmed of primer-binding sequences using Cutadapt and reads with no primer match were discarded^105^. Trimmed reads were then processed in USEARCH v11 to generate amplicon sequence variant (ASV) count tables^106^. Bacterial and Archaeal 16S gDNA reads were merged using the *fastq_mergepairs* command with maximum allowable mismatches in the overlapping region (*fastq_maxdiffs*) set to 20 and the minimum percent ID of alignment (*fastq_pctid*) left on the default setting of 90. The trimmed and merged reads were then quality-filtered using a maximum expected error threshold (*fastq*_*maxee*) of 1.0 and ASV denoising was conducted on dereplicated sequences using UNOISE3^107^. Singletons were removed and ASV tables were constructed with the *usearch_global* command using a similarity threshold of 99%. Since the denoising algorithm recovers the majority of true sequences in the sample, the 99% identity cut-off is fixed to allow for 1% error in the underlying reads believed to be generated by sequencing and PCR errors. Taxonomies for 16S rDNA ASVs were inferred from the silva_nr_132 reference database in Mothur v1.42.3^108,109^.

### Statistics and reproducibility

Unless otherwise stated, statistical analyses and additional data-processing steps were conducted in the R Statistical Environment and followed best practices for the handling of compositional data^110,111^. Imputation of zero-values was performed using Bayesian multiplicative replacement in the zCompositions package and read counts were converted to centred-log ratios (clr) prior to downstream analyses^112^. Patterns of microbial community assembly were assessed using non-metric multidimensional scaling (NMDS) based on Aitchison distance matrices calculated across samples using the vegan package^113^. The *envfit* function was used to test for significant effects of environmental parameters on microbial community dissimilarity. We considered dissolved inorganic nitrogen concentrations (NH_4_^+^, NO_3_^−^, NO_2_^−^), dissolved O_2_ concentrations, water column N_2_O saturations (ΔN_2_O), temperature, and salinity as potential predictors of microbial community structure.

Co-occurrence patterns between taxa with putative roles in N_2_O production and the rest of the microbial community ASVs were explored using proportionality analysis within the propr package^52^. ASV tables were trimmed to select taxa that occurred ≥10 times in at least 10% of samples prior to network-level analyses to improve interpretability and minimize the risk of spurious correlations. Pairwise interactions between individual taxa with rho values greater than 0.60 were plotted using Cytoscape v3.9.0 and network topological indices were calculated using the NetworkAnalyzer tool^114^. Relationships between microbial community structure and rate processes were then assessed using weighted gene correlational network analysis (WGCNA) performed with the WGCNA package^115^. The signed adjacency measure was first calculated for each pair of features (ASVs) by raising the absolute value of their pairwise correlation coefficients to a soft-thresholding power of 8 to maximize the scale-free topology fit. Hierarchical clustering of taxa into discrete subnetworks was completed using a minimum module size threshold of 20 and a dissimilarity threshold of 0.3. Pearson correlation coefficients and corresponding *p*-values are reported for correlations between sample traits, subnetwork eigengenes, and individual ASVs (Supplementary Data 1). Subnetwork membership and intranetwork connectivity measures are also reported for each ASV and were used in further analyses to assess broad relationships between ASV connectivity and importance with respect to N_2_O production rates.

Links between individual taxa and N_2_O production processes inferred from WGCNA were then confirmed using sparse partial least squares regression (sPLSR), implemented in the MixOmics package^116^. The advantage of sPLSR is that it is capable of modeling highly dimensional datasets with multiple noisy and collinear variables, making it a useful method for exploring relationships between two continuous datasets when the total number of variables greatly outnumbers the number of discrete observations^55^. This method combines dimension reduction and variable selection in a one-step modeling procedure, thus greatly improving interpretability over the standard PLSR approach. The final model was built after tuning based on Leave One Out cross-validation to determine the optimal number of latent components and variables for inclusion. Data sparsification is achieved by introducing a LASSO penalization to reduce the number of original variables used to construct the latent components. Pearson correlations between selected ASVs and sample traits were visualized in a clustered heatmap using a complete Euclidean distance method.

### Detecting putative keystone taxa

We explored the potential role of keystone taxa in mediating N_2_O production and accumulation by leveraging propr network topological indices to determine if taxa selected as important predictors of rate processes scored high on keystone measures. We considered high node (ASV) degree, closeness centrality, and betweenness centrality as indicators of microbial keystone taxa according to the recommendations of previous studies^28,53,54,117^. Whereas node degree represents the number of edges (associations) a particular ASV shares with others in the network, closeness centrality measures the average distance of each node to other nodes in the network. In contrast, betweenness centrality calculates the extent to which a particular node lies on the shortest path between two adjacent nodes^54^.

### Community functional gene predictions

Bacterial community functional compositions were predicted using PICRUSt2 version 2.4.1 with the default settings to assess relationships between predicted *nosZ* gene abundances and water column N_2_O saturations^118,119^. Briefly, ASVs were placed within a reference phylogeny based on 20,000 16S sequences from the Integrated Microbial Genomes database by multiple sequence alignment using HMMER (https://www.hmmer.org), optimal positioning of ASVs using EPA-ng^120^, and phylogenetic tree reconstruction using GAPPA^121^. The nearest-sequenced taxon index (NSTI) was calculated for each ASV and taxa with NSTI values less than 2.0 were excluded from downstream analyses. Less than 1% of bacterial ASVs (43 of 4674) were removed following quality filtering. Prediction of gene family abundances (including KEGG orthologues) was then conducted across samples using the Castor R package^122^. Predicted *nosZ* gene abundances were then selected from the model output based on the corresponding KEGG ortholog (K00376: nitrous-oxide reductase).

### Reporting summary

Further information on research design is available in the Nature Portfolio Reporting Summary linked to this article.

## Supplementary information

Supplementary Information

Description of Additional Supplementary Files

Supplementary Data 1-2

Reporting Summary

## Data Availability

All chemical and rate measurement data used in this study have been published previously in the Pangaea Repository at 10.1594/PANGAEA.912191^123^. Bacterial and archaeal 16S rRNA gene sequence data is accessible through the NCBI Sequence Read Archive (SRA) under BioProject number PRJNA901178. Results of network analyses and sPLSR used to construct Figs. 4 and 5 are contained in Supplementary Data 1 and Supplementary Data 2. Prokaryote ASV tables and predicted taxonomy files used to conduct statistical analyses and generate Figs. 1–3 can be accessed in the ‘Data’ folder at https://github.com/bdjameson/Interaction-networks under identifier 10.5281/zenodo.7604057^124^. PICRUSt2 predictions generated using Bacterial ASV sequences are contained in the same GitHub repository under subfolder ‘PICRUSt2’.

## Code availability

R code scripts used to process data and perform statistical analyses are available at https://github.com/bdjameson/Interaction-networks under identifier 10.5281/zenodo.7604057^124^.
