# Supplementary material for: Network analysis of 16S rRNA sequences suggests microbial keystone taxa contribute to marine N2O cycling
Source: Commun Biol. 2023 Feb 23;6:212. doi: 10.1038/s42003-023-04597-5 (PMC9950131; doi:10.1038/s42003-023-04597-5)
Supplement: Supplementary file 1 — Supplementary Information [file 42003_2023_4597_MOESM1_ESM.pdf]

Supplementary Information for:

Network analysis of 16S rRNA sequences suggests microbial keystone taxa contribute to marine N_2O cycling

Authors: *Brett D. Jameson [1], Sheryl A. Murdock [2,3], Qixing Ji [4], Catherine J. Stevens [1,2], Damian S. Grundle [3,5], S. Kim Juniper [1,2,6]

Author affiliations:

1. School of Earth & Ocean Sciences, University of Victoria, P.O. Box 1700 Station CSC, Victoria, British Columbia, V8W 2Y2, Canada

2. Department of Biology, University of Victoria, P.O. Box 1700 CSC, Victoria, British Columbia, V8W 2Y2, Canada

3. Bermuda Institute of Ocean Sciences, 17 Biological Station, St. George's, GE01, Bermuda.

4. Thrust of Earth, Ocean & Atmospheric Sciences, Hong Kong University of Science and Technology (Guangzhou), Nansha, Guangzhou, Guangdong, 511400, China.

5. School of Ocean Futures & School of Earth & Space Exploration, Arizona State University, Tempe, Arizona, USA, 85287-7904.

6. Ocean Networks Canada, 2474 Arbutus Road, Victoria, BC, V8N 1V8, Canada

Correspondence to: Brett D. Jameson (bjameson@uvic.ca).

Contents:

Supplementary Table 1. Primer sequences used for 16S rRNA sequencing 2

Supplementary Table 2. Bacterial and archaeal 16S rRNA amplicon read counts 2

Supplementary Figure 1. Map of sampling location in Saanich Inlet, B.C. Canada 3

Supplementary Figure 2. Relative abundances of AOA and NOB calculated across samples.................. 4

Supplementary Figure 3. Results of SPLSR analyses with ASV identifiers 5

Supplementary Figure 4. N_2O production from NH_4^+ oxidation scatterplots... 6

Supplementary Figure 5. Nitrification rates versus *in situ* O_2 and NH_4^+ concentrations 6

Supplementary Tables:

Supplementary Table 1. Primer sequences used to target the V6-V8 variable regions of the bacterial and archaeal 16S rRNA gene for high-throughput sequencing on Illumina MiSeq at the Integrated Microbiome Resource at Dalhousie University (https://imr.bio/protocols.html).

Primer sequences	Forward Primer (5’-3’)	Reverse Primer (5’-3’)
Bacteria (B969F/BA1406R)	ACGCGHNRAACCTTACC	ACGGGCRGTGWGTRCAA
Archaea (A956F/A1401R)	TYAATYGGANTCAACRCC	CRGTGWGTRCAAGGRGCA

Supplementary Table 2. Total read counts reported for bacterial and archaeal 16S rRNA gene amplicon reads in each sample following quality control and paired-end read merging. Depths and dates of sampling are recorded in addition to sample IDs.

Sample ID	Date	Depth	Bacteria	Archaea
APR_75	05-Apr-18	75	20125	8416
APR_90	05-Apr-18	90	17675	16455
APR_100	05-Apr-18	100	16185	15640
APR_110	05-Apr-18	110	19691	10423
APR_130	05-Apr-18	130	19928	8940
APR_160	05-Apr-18	160	12103	14636
JUN_75	14-Jun-18	75	15136	NA
JUN_90	14-Jun-18	90	14149	NA
JUN_100	14-Jun-18	100	16442	9224
JUN_110	14-Jun-18	110	15668	4873
JUN_130	14-Jun-18	130	26512	9230
JUN_160	14-Jun-18	160	19616	NA
AUG_75	02-Aug-18	75	9268	NA
AUG_90	02-Aug-18	90	7160	NA
AUG_100	02-Aug-18	100	10180	4271
AUG_110	02-Aug-18	110	10516	4391
AUG_130	02-Aug-18	130	21470	6912
AUG_160	02-Aug-18	160	7322	NA
OCT_75	25-Oct-18	75	13483	18683
OCT_90	25-Oct-18	90	13754	13005
OCT_100	25-Oct-18	100	25608	16961
OCT_110	25-Oct-18	110	17409	10915
OCT_130	25-Oct-18	130	12452	22826
OCT_160	25-Oct-18	160	15320	8355

Supplementary Figures:

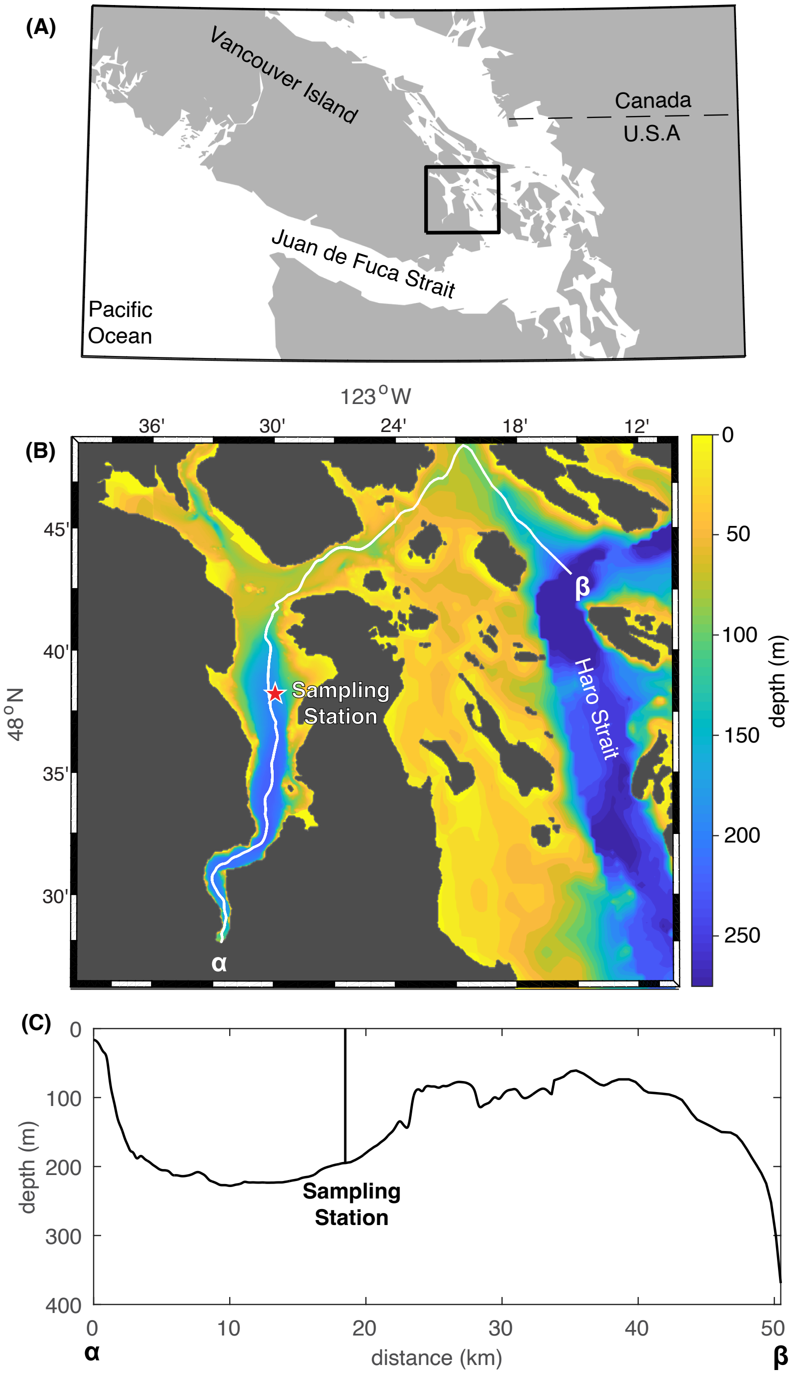

Supplementary Figure 1. Map of Saanich Inlet, B.C., Canada (a) including bathymetric cross section traversing the central deep basin and shallow sill located at Satellite Channel (b). Cross section in panel b follows the dashed transect line depicted in panel a. Sampling location is indicated by a star in both panels (48°47.707'N, 123°29.927'W). Figure adapted from Soetaert et al. (2020) by Roberta C. Hamme (Ref. 1).

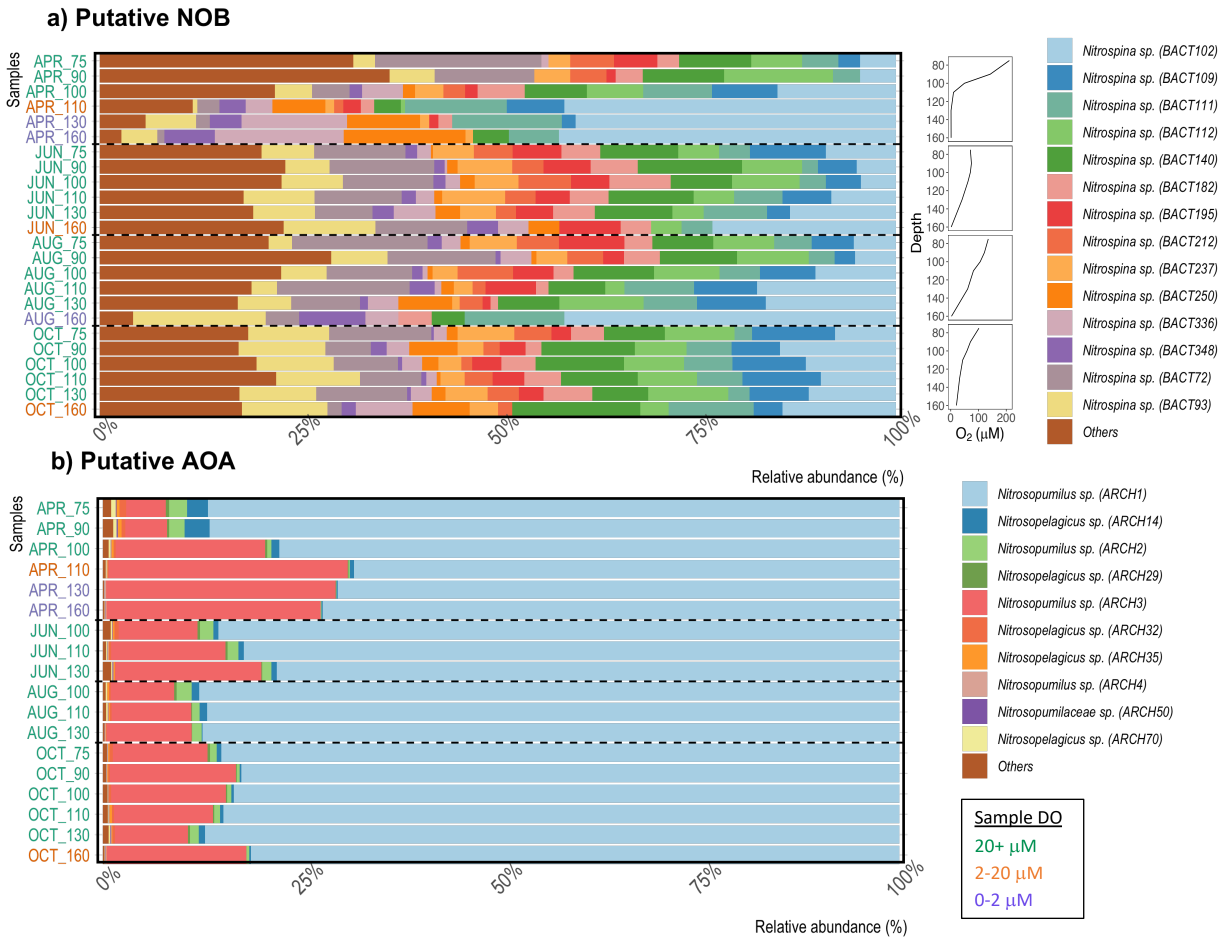

Supplementary Figure 2. Relative abundances of a) putative nitrite-oxidizing Bacteria (NOB) and b) putative ammonium-oxidizing Archaea (AOA) ASVs in seston samples obtained in Saanich Inlet. Dissolved oxygen profiles for each sampling period are reported in panel a). Samples were obtained from Saanich inlet between April and October 2018. Sample labels on the vertical axes correspond to sampling month and water column depth.

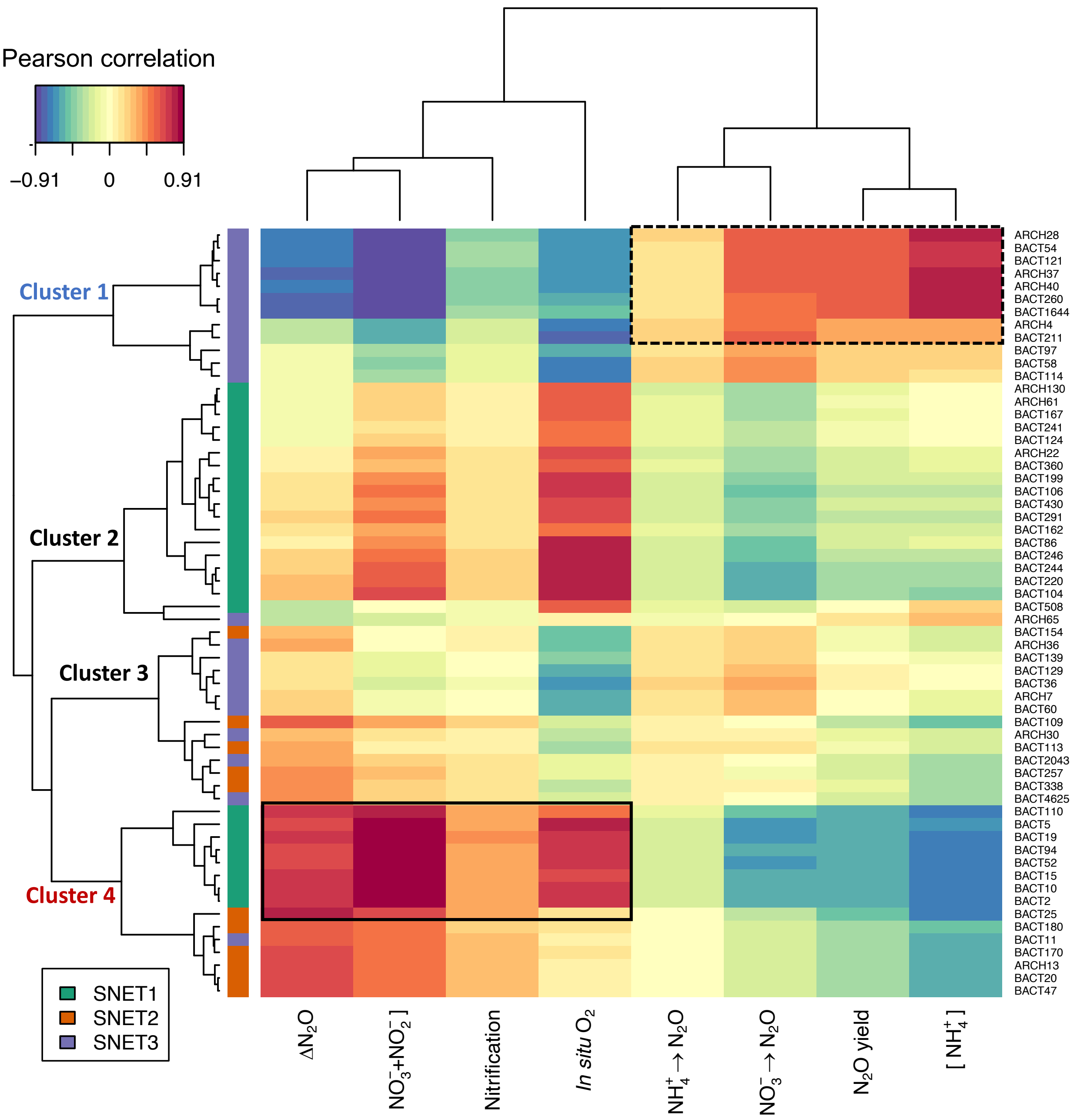

Supplementary Figure 3. Relationships between prokaryotic amplicon sequence variants (ASVs), relevant environmental variables and process rates. N_2O production rates from NH_4^+ oxidation and NO_3^- reduction are symbolized by black arrows. Pairwise correlation coefficients between ASVs and sample traits were calculated using a two-component sPLS regression model and are presented as a clustered heatmap. Taxa with correlations to nitrification rates > 0.30 are indicated by solid black lines and taxa with correlations to N_2O production from NO_3^- reduction > 0.50 are indicated by dashed lines. Hierarchical clustering of variables was achieved using a complete Euclidean distance method. ASV subnetwork assignments determined through WGCNA are indicated by coloured rectangles on the vertical axis dendrogram. This figure was reproduced from Fig. 5 in the main text but replaces row names with microbial ASV identifiers.

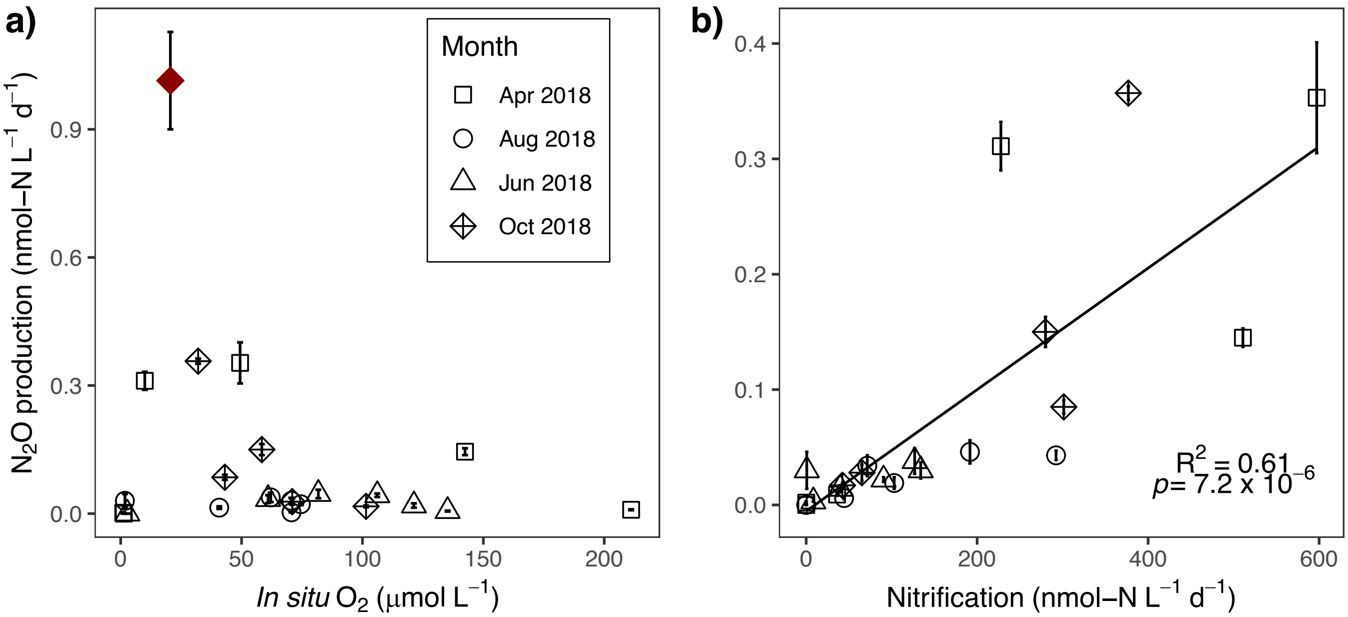

Supplementary Figure 4. Scatterplots showing relationships between rates of N_2O production from NH_4^+ oxidation (nmol-N L^{-1} d^{-1}) and a) *in situ* dissolved O_2 concentrations (umol L^{-1}) and b) nitrification rates (nmol-N L^{-1} d^{-1}). Linear regression in panel B was conducted following removal of the outlier point highlighted in red in panel A. Reproduced from data reported in Ji et al. (2020) (Ref. 2). Error bars represent ± SD (n = 5).

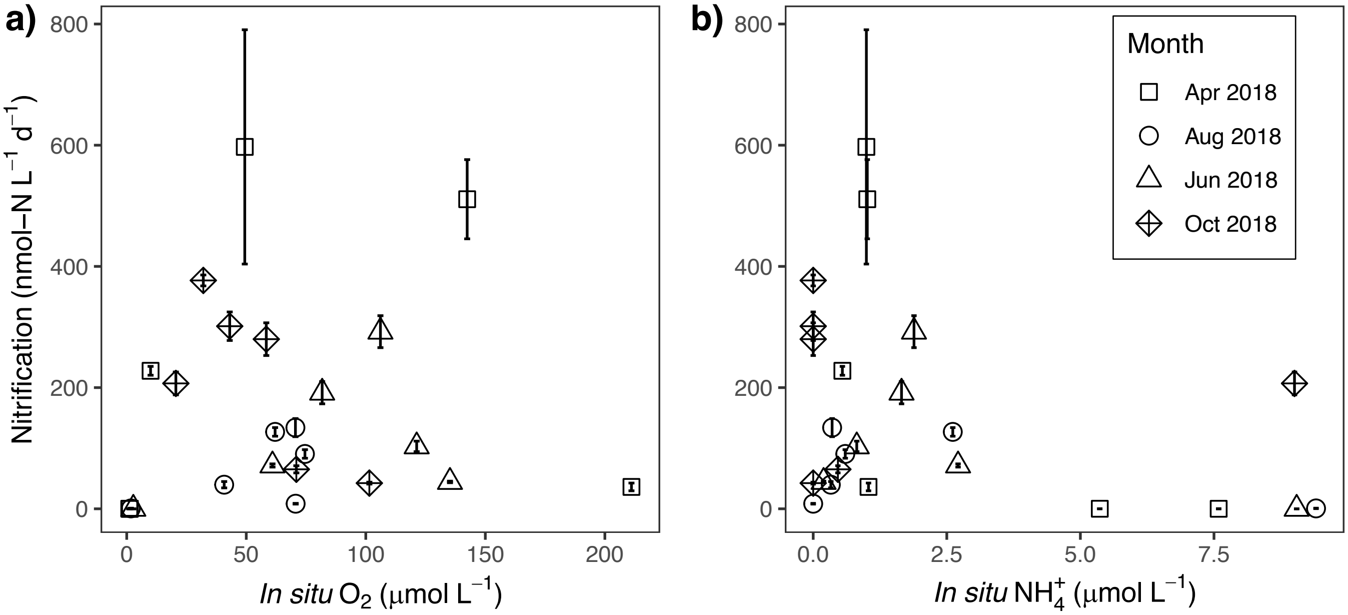

Supplementary Figure 5. Scatterplots showing relationships between nitrification rates (nmol-N L^{-1} d^{-1}) and a) *in situ* dissolved O_2 concentrations (umol L^{-1}) and b) *in situ* dissolved NH_4^+ concentrations (umol L^{-1}). Reproduced from data reported in Ji et al. (2020) (Ref. 2). Error bars represent ± SD (n = 5).

Supplementary References:

1. Soetaert, G., Hamme, R. C., & Raftery, E. (2022). Renewal of seasonally anoxic Saanich Inlet is temporally and spatially dynamic. *Fronteris in Marine Science* 9:1001146. https://doi.org/10.3389/fmars.2022.1001146
2. Ji, Q., Jameson, B. D., Juniper, S. K., & Grundle, D. S. (2020). Temporal and Vertical Oxygen Gradients Modulate Nitrous Oxide Production in a Seasonally Anoxic Fjord: Saanich Inlet, British Columbia. *Journal of Geophysical Research: Biogeosciences*, 125(9), e2020JG005631. https://doi.org/10.1029/2020JG005631
