# Supplementary material for: Network analysis of 16S rRNA sequences suggests microbial keystone taxa contribute to marine N2O cycling
Source: Commun Biol. 2023 Feb 23;6:212. doi: 10.1038/s42003-023-04597-5 (PMC9950131; doi:10.1038/s42003-023-04597-5)
Supplement: Supplementary file 2 — Description of Additional Supplementary Files [file 42003_2023_4597_MOESM2_ESM.pdf]

Description of Additional Supplementary Files

File name: Supplementary Data 1
Description: Source data behind Figure 4.

File name: Supplementary Data 2
Description: Source data behind Figure 5.
