# Supplementary material for: Network analysis of 16S rRNA sequences suggests microbial keystone taxa contribute to marine N2O cycling
Source: Commun Biol. 2023 Feb 23;6:212. doi: 10.1038/s42003-023-04597-5 (PMC9950131; doi:10.1038/s42003-023-04597-5)
Supplement: Supplementary file 4 — Reporting Summary [file 42003_2023_4597_MOESM4_ESM.pdf]

Corresponding author(s): Brett D. Jameson
Last updated by author(s): Feb 2, 2023

Reporting Summary

Nature Portfolio wishes to improve the reproducibility of the work that we publish. This form provides structure for consistency and transparency in reporting. For further information on Nature Portfolio policies, see our Editorial Policies and the Editorial Policy Checklist.

Statistics

For all statistical analyses, confirm that the following items are present in the figure legend, table legend, main text, or Methods section.

n/a	Confirmed	
☒	☐	The exact sample size (*n*) for each experimental group/condition, given as a discrete number and unit of measurement
☐	☒	A statement on whether measurements were taken from distinct samples or whether the same sample was measured repeatedly
☐	☒	The statistical test(s) used AND whether they are one- or two-sided<br>*Only common tests should be described solely by name; describe more complex techniques in the Methods section.*
☐	☒	A description of all covariates tested
☒	☐	A description of any assumptions or corrections, such as tests of normality and adjustment for multiple comparisons
☐	☒	A full description of the statistical parameters including central tendency (e.g. means) or other basic estimates (e.g. regression coefficient) AND variation (e.g. standard deviation) or associated estimates of uncertainty (e.g. confidence intervals)
☒	☐	For null hypothesis testing, the test statistic (e.g. *F*, *t*, *r*) with confidence intervals, effect sizes, degrees of freedom and *P* value noted<br>*Give P values as exact values whenever suitable.*
☒	☐	For Bayesian analysis, information on the choice of priors and Markov chain Monte Carlo settings
☒	☐	For hierarchical and complex designs, identification of the appropriate level for tests and full reporting of outcomes
☐	☒	Estimates of effect sizes (e.g. Cohen's *d*, Pearson's *r*), indicating how they were calculated

Our web collection on statistics for biologists contains articles on many of the points above.

Software and code

Policy information about availability of computer code

Data collection	CTD profile data (conductivity, temperature, depth, dissolved oxygen) was collected using Seabird Seasoft V2.4.0. Sample N2O concentrations and isotopic ratios used to calculate production rates were collected on a Picarro G5131-i isotopic and gas concentration analyzer with built-in software. Bacterial and archaeal 16S rRNA genes were selected for sequencing from raw extracts using universal primer sets targeting the V6-V8 variable regions on an Illumina MiSeq at the Integrated Microbiome Resource
Data analysis	Unless otherwise stated, statistical analyses and additional data-processing steps were conducted in the R Statistical Environment (R Core Team, 2021). Species co-occurence patterns were explored using proportionality analysis within the 'propr' package (Quinn et al., 2017) and visualized using Cytoscape v3.9.0 (Shannon et al., 2003). Network topological indices used to infer keystone taxa were caclulated using the NetworkAnalyzer tool within the Cytoscape software. Weighted gene correlational network analyses were employed via the 'WGCNA' package (Langfelder and Horvath, 2008) to delineate microbial community subnetworks, estimate connectivity measures for each ASV and explore correlations between microbial community structure and N2O production proxies. Taxon-specific correlations between putative keystone taxa N2O production proxies were explored using a sparse partial least-squares regression (sPLSR), implemented in the 'MixOmics' package (Rohart et al. 2017).

For manuscripts utilizing custom algorithms or software that are central to the research but not yet described in published literature, software must be made available to editors and reviewers. We strongly encourage code deposition in a community repository (e.g. GitHub). See the Nature Portfolio guidelines for submitting code & software for further information.

Data

Policy information about availability of data

All manuscripts must include a data availability statement. This statement should provide the following information, where applicable:

- Accession codes, unique identifiers, or web links for publicly available datasets
- A description of any restrictions on data availability
- For clinical datasets or third party data, please ensure that the statement adheres to our policy

All chemical and rate measurement data used in this study have been published previously in the Pangaea Repository at https://doi.pangaea.de/10.1594/PANGAEA.912191. All 16S rRNA gene sequence data are available through the NCBI Sequence Read Archive under BioProject ID PRJNA901178. Additional data files and R code are available at https://github.com/bdjameson/Interaction-networks.

Human research participants

Policy information about studies involving human research participants and Sex and Gender in Research.

Reporting on sex and gender	NA
Population characteristics	NA
Recruitment	NA
Ethics oversight	NA

Note that full information on the approval of the study protocol must also be provided in the manuscript.

Field-specific reporting

Please select the one below that is the best fit for your research. If you are not sure, read the appropriate sections before making your selection.

☐ Life sciences ☐ Behavioural & social sciences ☒ Ecological, evolutionary & environmental sciences

For a reference copy of the document with all sections, see nature.com/documents/nr-reporting-summary-flat.pdf

Ecological, evolutionary & environmental sciences study design

All studies must disclose on these points even when the disclosure is negative.

Study description

This study involved a six-month time series of water column observations in Saanich Inlet, a seasonally anoxic fjord located on Vancouver Island, Canada, aimed at identifying the relationships between microbial community structure and variability in water column N2O cycling. We leverage fine-resolution molecular marker profiling of prokaryotic communities, community-level network analyses, and multivariate statistical modelling to identify patterns of niche-differentiation and link community dynamics to N2O production and accumulation across vertical and temporal redox gradients. Sampling was conducted bi-monthly at standardized depths selected to encompass a broad range of water column O2 and dissolved inorganic nitrogen species concentrations, as well as N2O saturation values. Rates of N2O production from NH4 oxidation (nitrification) and NO3- reduction (denitrification) were quantified at each depth to discern dominant production pathways.

Research sample

At each sampling date, seston samples were collected from six discrete depths across the redox boundary for total microbial DNA in order to characterize variability in microbial community structure and network assembly. Additional samples were obtained at each depth for quantification of dissolved inorganic nitrogen (NO3-, NO2-, NH4+) and N2O concentrations, as well as N2O production rates from discrete pathways. The specifics of the chemical analyses and 15N-labeled tracer experiments to measure N2O production rates from NH4+ oxidation and NO3- reduction, have been detailed in Ji et al.(2020) (https://doi.org/10.1029/2020JG005631).

Sampling strategy

Seston samples for DNA extraction and sequencing were obtained immediately following water column sampling by filtering 5 L of seawater onto 0.2 µm Sterivex filters (Merck) by peristalsis. A single 5 L sample was obtained at each depth to represent microbial community composition. Previous studies have demonstrated that this is a sufficient volume for high-yield extraction of total microbial DNA. Microscopic cell counts revealed that 5 L of seawater from these depths and at this location contain roughly 5 x 10^8 prokaryotic cells (Bacteria and Archaea) (Hamme et al., in prep) and thus providing a large mixed sample that adequately represents anticipated microbial community composition.

Data collection

Field collection of samples was conducted by Brett Jameson (corresponding author) and Qixing Ji (co-author). Water column CTD profiles were conducted at each sampling date by Brett Jameson and Qixing Ji to obtain full water column profiles of temperature, salinity and dissolved oxygen. Dissolved inorganic nitrogen and N2O concentrations were measured by Qixing Ji as follows: NH4+ was measured fluorometrically by reaction with orthophthaldialdehyde, NO2- was measured colorimetrically following treatment of samples with Greiss reagent, NO3- + NO2- was measured using the cadmium reduction method, and N2O concentrations were measured using a purge-trap module coupled to a cavity ring-down spectrometer (PT-CRDS) (Ji et al., 2020). DNA was extracted from

	seawater samples by Brett Jameson and cleaned extracts were sent for amplicon sequencing at the Integrated Microbiome Resource at Dalhousie University (https://imr.bio/index.html).
Timing and spatial scale	Field sampling was conducted on four separate dates at a single sampling location in the Saanich Inlet deep basin. Specific sampling dates were 5 April, 14 June, 02 August, and 25 October 2018. For each sampling date, field operations were conducted between 07:30 and 17:00. Seawater incubations to measure N2O production rates were conducted immediately following field operations in a nearby lab and lasted 12-18 hours. Samples taken for measurement of dissolved inorganic nitrogen and N2O concentrations were preserved (flash frozen and stored at -20 C for DIN, mercuric chloride preservation for N2O) and analyzed within 4 months of initial sampling. DNA samples were flash frozen and stored at -80C before extraction within 6-12 months of initial sampling.
Data exclusions	Sequencing of archaeal 16S genes yielded data for 18 of 24 samples while sequencing of the bacterial 16S gene yielded data for all 24 samples. As a result, network-level analyses involving the combined bacterial and archaeal datasets include samples that yielded data for both bacteria and archaea. However, results of the network analyses were robust to re-analysis using only the bacterial ASV tables and we do not believe our results or interpretations to be impacted as a result of these exclusions.
Reproducibility	Data analyses were automated and corresponding code scripts as well as all data and metadata files necessary to reproduce the findings presented have been published in a public repository. For pre-processing of amplicon sequence data, all quality control and filtering parameters, thresholds for removal of rare taxa, as well as details for data transformations have been disclosed in the methods. All DNA sequences generated are available through the Sequences Read Archive under BioProject ID PRJNA901178.
Randomization	This study did not involve experimental manipulations of microbial communities and thus randomization is not relevant.
Blinding	Blinding is not relevant to this study as the study design did not involve experimental manipulation or different experimental treatments.

Did the study involve field work? ☒ Yes ☐ No

Field work, collection and transport

Field conditions	Water column sampling was conducted exclusively below 75 m depth, well below the mixed layer in Saanich Inlet. As a result, field conditions at the surface did not impact conditions at our sampling depths.
Location	Saanich Inlet, Vancouver Island, Canada (48° 37.53’N, 123° 29.91’W). Sampling depths across all dates were set at 75, 90, 100, 110, 130, and 160 m.
Access & import/export	Sampling was conducted on the University of Victoria's MSV Strickland using a CTD rosette deployed off the stern from the ship's A-frame. DNA and nutrients samples were flash frozen on dry ice and transported in a cooler to the University of Victoria immediately following field operations where they were stored until laboratory analysis. N2O samples were shipped to the Bermuda Institute of Ocean Sciences via FedEx courier services for analysis on the PT-CRDS system.
Disturbance	There were no notable disturbances caused during the conduction of this study.

Reporting for specific materials, systems and methods

We require information from authors about some types of materials, experimental systems and methods used in many studies. Here, indicate whether each material, system or method listed is relevant to your study. If you are not sure if a list item applies to your research, read the appropriate section before selecting a response.

Materials & experimental systems

n/a	Involved in the study
☒	☐ Antibodies
☒	☐ Eukaryotic cell lines
☒	☐ Palaeontology and archaeology
☐	☒ Animals and other organisms
☒	☐ Clinical data
☒	☐ Dual use research of concern

Methods

n/a	Involved in the study
☒	☐ ChIP-seq
☒	☐ Flow cytometry
☒	☐ MRI-based neuroimaging

Animals and other research organisms

Policy information about studies involving animals; ARRIVE guidelines recommended for reporting animal research, and Sex and Gender in Research

Laboratory animals	This study did not involve laboratory animals

Wild animals	This study did not involve wild animals
Reporting on sex	Sampling of biological organisms involved microbes only
Field-collected samples	Field sampling of biological organisms involved microbes only. These samples were flash-frozen immediately following sampling and stored at -80 C prior to DNA extraction.
Ethics oversight	Field sampling of biological organisms involved microbes only, no ethics approval was necessary

Note that full information on the approval of the study protocol must also be provided in the manuscript.
